# Over-the-Scope Clip for Treating Chronic Bariatric Surgery Leaks Refractory to Prior Endoscopic Therapies: Experience From a Bariatric Center of Excellence

**DOI:** 10.7759/cureus.101723

**Published:** 2026-01-17

**Authors:** Talal K Khewater, Husaam Adi, Ahmad S Alayed, Abdulaziz S Alharthi, Abdulwahab N Aladhyani

**Affiliations:** 1 Department of Surgery, King Salman Armed Forces Hospital, Tabuk, SAU

**Keywords:** bariatric surgery, chronic leak, endoscopic closure, over-the-scope clip (otsc), ovesco, refractory leak

## Abstract

Background

Chronic leaks following bariatric surgery continue to present significant therapeutic challenges, particularly when conventional endoscopic interventions are unsuccessful. The over-the-scope clip (OTSC) is increasingly being employed as a salvage approach; however, supporting evidence remains limited. This study sought to assess the efficacy of the OTSC in complex cases in which conventional endoscopic interventions proved unsuccessful. We reviewed all refractory cases and proposed the use of the clip as an innovative strategy for these situations.

Methods

We performed a retrospective analysis of six consecutive patients with chronic post-bariatric leaks unresponsive to standard endoscopic therapy who underwent OTSC placement at our center of excellence between May 2022 and October 2025. Data on patient demographics, prior interventions, technical and clinical outcomes, and follow-up were collected and analyzed.

Results

All six patients (mean age 39.7 ± 11.9 years; BMI 44.0 ± 5.2 kg/m²; three males, three females) had previously undergone either primary or revisional bariatric procedures. Prior endoscopic interventions included fully covered self-expandable metallic stents, internal drainage, and septotomy, with several patients requiring multiple surgical sessions for lavage and drainage. OTSC placement was technically successful in all cases (100%), with clinical closure achieved in five of six patients (83.3%) over a mean follow-up of 525.2 ± 413.9 days (range, 93-1221 days). One patient who did not achieve closure required additional endoscopic therapy and multiple subsequent surgical interventions. No adverse events or mortality were reported.

Conclusions

OTSC placement is a feasible and effective salvage therapy for chronic bariatric surgery leaks refractory to conventional endoscopic approaches. Our findings show high technical success and favorable clinical outcomes, with most patients achieving durable leak closure and no significant adverse events. The absence of complications or mortality further supports the safety profile of this approach in carefully selected patients. In the interim, OTSC should be considered a valuable tool in the multidisciplinary management of refractory post-bariatric leaks, offering the potential for improved outcomes in this complex patient population.

## Introduction

Leaks after bariatric surgery represent one of the most serious complications, with an incidence ranging from 0.7% to 5.7%, depending on the procedure [1-4]. Chronic leaks are typically defined as those occurring beyond 12 weeks (84 days) after the initial surgery [5]. In contrast, leaks that develop earlier but persist despite comprehensive intervention currently lack a well-established definition. These persistent fistulas are particularly challenging to manage due to fibrosis, established fistula tracts, and patient morbidity [6,7]. Conventional endoscopic techniques, such as fully covered self-expandable metallic stents (SEMSs), internal drainage, and tissue sealants, often fail in this subgroup [8-10].

The over-the-scope clip (OTSC) system allows full-thickness closure of gastrointestinal wall defects and has been increasingly applied to post-surgical leaks [11,12]. While promising, most available reports focus on acute or subacute leaks, and evidence for chronic, refractory cases remains limited [13].

We present our institutional experience with OTSC in a series of six patients with chronic post-bariatric leaks unresponsive to conventional endoscopic therapy.

This article was previously posted to In Review, © Springer Nature, preprint server, on November 15, 2025.

## Materials and methods

Study design and patients

This retrospective case series included all consecutive patients treated with OTSC (Ovesco Endoscopy AG, Tübingen, Germany) for chronic post-bariatric leaks at King Salman Armed Forces Hospital, Tabuk, Saudi Arabia, “The world’s first SRC-accredited center of excellence in metabolic and bariatric revisional surgery” [14], between May 2022 and August 2025. Chronic leak was defined as persistence beyond eight weeks (56 days) despite prior interventions. Patients who responded to any endoscopic or surgical interventions within 56 days were excluded.

Data collection

Clinical data were extracted from electronic medical records and included demographics, BMI at the time of leak surgery, leak site, interval from initial surgery to leak, prior endoscopic procedures, time to OTSC placement, and OTSC size and type used [15].

Outcomes

Technical success was defined as successful OTSC deployment at the target site. Clinical success was defined as leak closure accompanied by symptom resolution and radiologic confirmation during follow-up. Secondary outcomes included the need for additional therapy, adverse events, and mortality.

Statistical analysis

Continuous variables are expressed as mean ± SD or median (range), and categorical variables as frequencies and percentages.

## Results

Patient characteristics

Six patients were included (three males, three females; mean age 39.7 ± 11.9 years; mean BMI 44.0 ± 5.2 kg/m²). Two patients (33.3%) underwent primary sleeve gastrectomy (SG), and four (66.7%) had conversional surgeries. Patient demographics are summarized in Table 1.

**Table 1:** Patient demographics (n = 6)

Variable	n (%) or mean ± SD
Number of patients	6
Age (years)	39.7 ± 11.9
Sex (male/female)	3/3
BMI before leak (kg/m²)	44.0 ± 5.2
Type of surgery leading to a leak	
Primary	2 (33.3%)
Conversional	4 (66.7%)

Leak details and prior therapy

**Figure 1:**
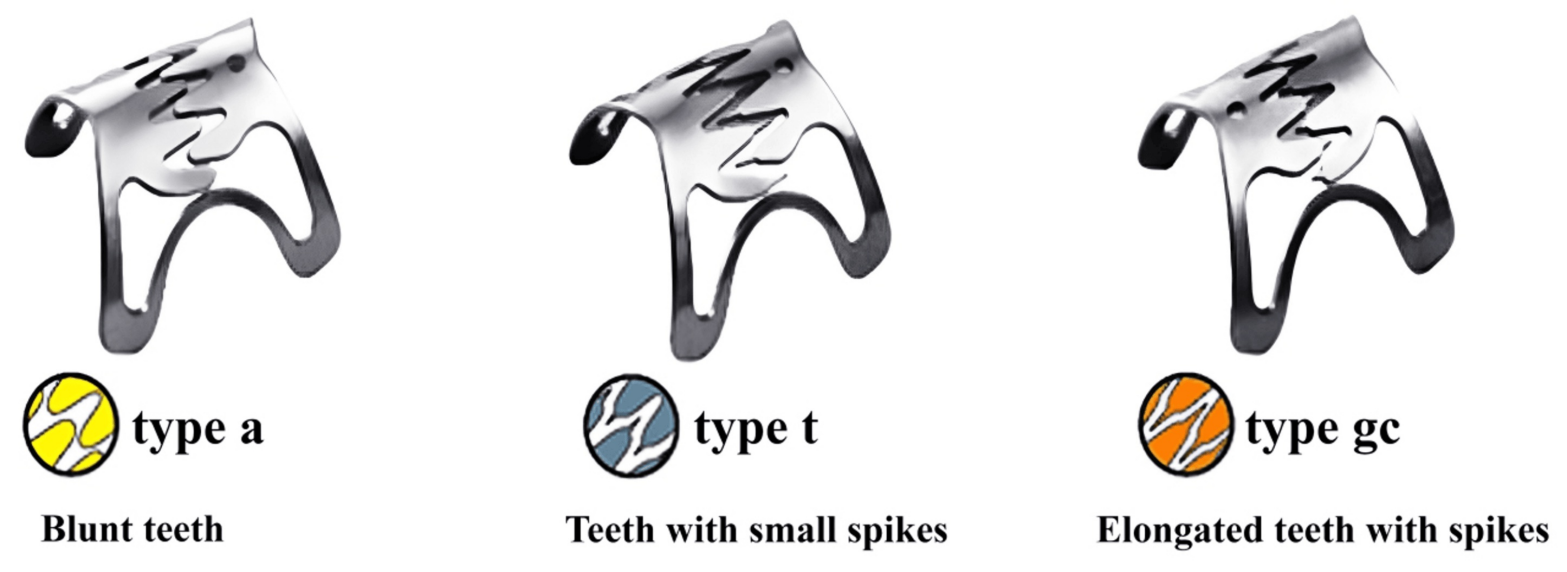
Types of OTSC OTSC, over-the-scope clip Source: [16]

Leaks occurred two to 21 days postoperatively in all patients and were located at the angle of His, gastro-gastric anastomosis, or gastrojejunostomy. All patients had previously failed treatment with stents or drains. One patient underwent multiple interventions and did not achieve leak closure with OTSC. Additional details are provided in Table 2, and Figure 1 shows the types and sizes of OTSC used.

**Table 2:** Clinical details and management of leaks (n = 6) Size/type codes correspond to Figure 1. GJ, gastrojejunostomy; OAGB, one-anastomosis gastric bypass; OTSC, over-the-scope clip; RYGB, Roux-en-Y gastric bypass; SEMS, self-expandable metallic stent; SG, sleeve gastrectomy; VBG, vertical banded gastroplasty

Patient	Type of surgery	Time to leak (days)	Leak site	Prior endoscopic maneuvers	Time to OTSC (days)	OTSC size/type
1	Primary SG	21	Angle of His	SEMS × 50 days	89	(2) 11/6t
2	Primary SG	17	Angle of His	SEMS × 39 days	61	(1) 12/6gc
3	Conversion RYGB → SG	7	Gastro-gastric anastomosis	SEMS × 49 days	57	(1) 12/6gc
4	Conversion RYGB → SG	8	Gastro-gastric anastomosis	SEMS × 71 days	86	(1) 12/6t
5	Conversion VBG → RYGB	21	Gastrojejunal (GJ) anastomosis	SEMS × 42 days	130	(1) 12/6gc
6	SG → OAGB → RYGB	2	Angle of His and GJ anastomosis	Double pigtail drain × 120 days → SEMS × 37 days → OTSC × 2 trials	170-200	(3) 12/6gc

Outcomes

OTSC deployment was technically successful in all cases (100%). Clinical closure was achieved in five of six patients (83.3%), with a mean follow-up of 525.2 ± 413.9 days (range, 93-1221 days). One patient required additional endoscopic interventions and subsequently underwent surgical treatment. No adverse events or mortalities were reported. Further details are provided in Table 3.

**Table 3:** Outcomes after OTSC for chronic post-bariatric leaks (n = 6) OTSC, over-the-scope clip

Outcome	n (%) or mean ± SD
Technical success	6/6 (100%)
Clinical closure	5/6 (83.3%)
Follow-up	Mean 525.2 ± 413.9 days (93-1221)
Additional endoscopy	1/6 (16.7%)
Surgical re-intervention	1/6 (16.7%)
Adverse events	None
30-day mortality	0/6 (0%)

## Discussion

Chronic fistulas following bariatric surgery are complex and pose significant therapeutic challenges. A range of strategies has been described in the literature, including surgical drainage, image-guided procedures, and endoscopic methods such as pigtail drain insertion, endoprosthesis placement, clipping, and the use of sealing agents, including glue or fibrin sealants. When endoscopic management fails, definitive surgical options are recommended, including gastrojejunal anastomosis, Roux-en-Y gastric bypass (RYGB), gastrectomy with esophagojejunal anastomosis, and other approaches [17,18].

We present an alternative approach using OTSC for the management of complex, persistent fistulas after unsuccessful conventional endoscopic interventions. Although the patient cohort is small, these findings may support further investigation in this field.

Among six patients with chronic post-bariatric leaks unresponsive to various endoscopic therapies, OTSC application achieved a 100% technical success rate and an 83.3% clinical success rate. These results are consistent with published literature reporting efficacy rates of 70-90% [12,13].

**Figure 2:**
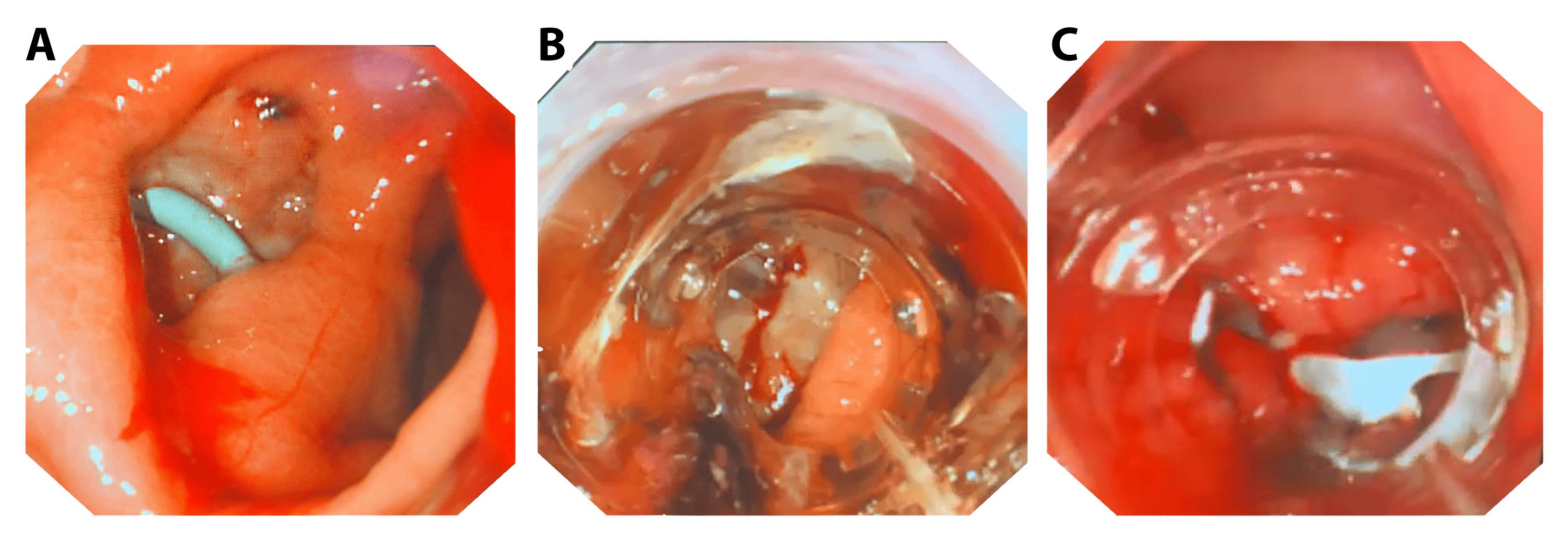
Endoscopy view: illustration of the OTSC deployment (A) Fistula demonstrating the presence of a drain during stent removal. (B) OTSC positioned prior to deployment. (C) OTSC in place following deployment. OTSC, over-the-scope clip

**Figure 3:**
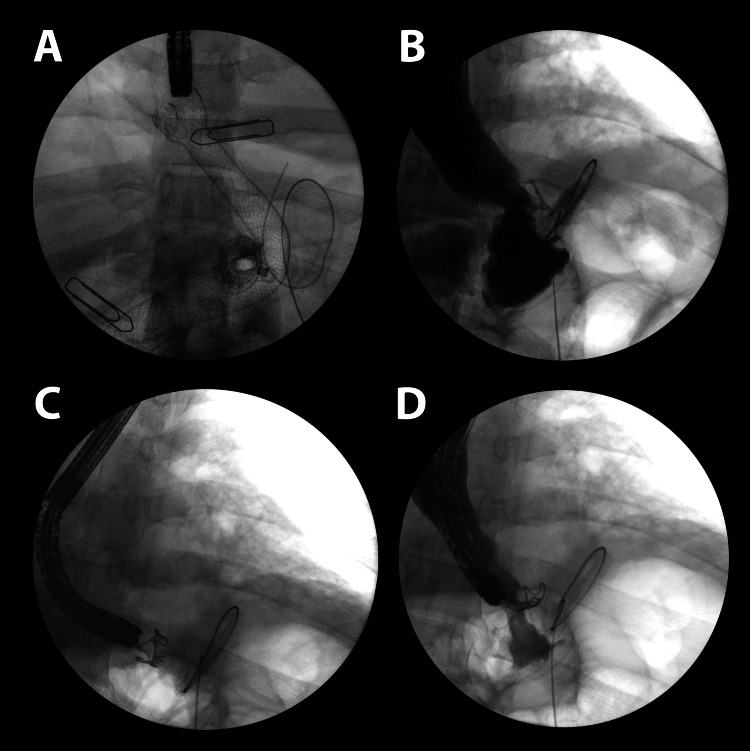
Fluoroscopy view: illustration of the OTSC positioning (A) SEMS prior to removal. (B) Evidence of leakage following SEMS removal. (C) OTSC positioned pre-deployment. (D) OTSC post-deployment with no observed leakage. OTSC, over-the-scope clip; SEMS, self-expandable metallic stent

Both patients with leaks following SG were initially managed with fully covered SEMSs maintained for 50 and 39 days, respectively. Persistent fistulas at the time of stent removal were subsequently treated with OTSC clips (Figure 2, Figure 3).

**Figure 4:**
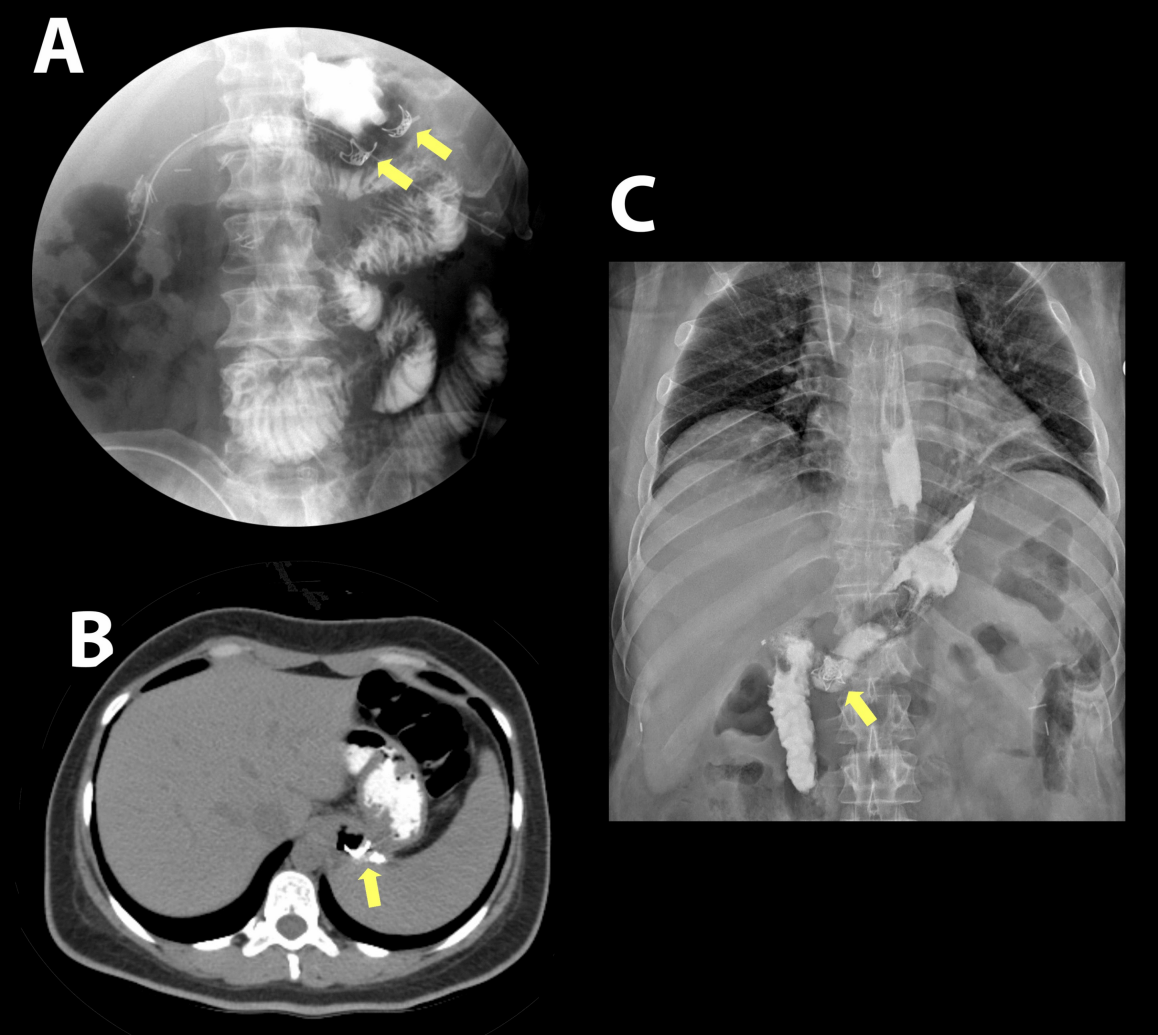
Follow-up imaging (A) Fluoroscopic image showing two OTSCs with Gastrografin, demonstrating no leakage. (B) Contrast-enhanced CT scan. (C) Radiograph depicting OTSC in situ. OTSC, over-the-scope clip

In two patients who underwent conversion from RYGB to SG due to weight regain and hypoglycemia, SEMS were placed for more than eight weeks to address gastro-gastric anastomotic leaks. Following stent removal, persistent fistulas were identified radiologically, prompting the use of OTSCs, which resulted in complete closure (Figure 4).

The patient who developed a leak after conversion from vertical banded gastroplasty to RYGB received an OTSC at the gastrojejunal anastomosis following failure of SEMS management over 42 days.

Another patient, who had previously undergone conversion from SG to one-anastomosis gastric bypass at another institution, experienced a postoperative leak on day two. Despite subsequent conversion to RYGB and multiple endoscopic interventions, including double pigtail internal drainage and SEMS placement, persistent leaks were noted at both the angle of His and the gastrojejunal anastomosis four months later. Multiple OTSC applications were unsuccessful in resolving the complex tract leak, ultimately leading to completion gastrectomy with esophagojejunal anastomosis.

Long-term follow-up exceeding one year in selected cases demonstrated the sustained efficacy of OTSC, consistent with previous studies reporting durable outcomes [19,20]. No adverse events were observed, further supporting the favorable safety profile of OTSC in this setting. Figure 2, Figure 3, and Figure 4 provide representative examples of the procedures performed.

This study has several limitations, including its retrospective design, small sample size, and single-center scope. To establish an effective management algorithm for persistent post-bariatric leaks using OTSC, further multicenter studies are warranted. Nonetheless, OTSC appears safe and effective as a rescue therapy for leaks unresponsive to conventional endoscopic treatments, achieving high success rates and durable clinical benefits.

## Conclusions

OTSC placement is a viable and effective salvage therapy for chronic bariatric surgery leaks unresponsive to conventional endoscopic techniques. The results demonstrate high technical success and favorable clinical outcomes, with most patients achieving sustained leak closure without significant adverse events. The absence of complications or mortality underscores the safety profile of OTSC in appropriately selected cases. OTSC should be regarded as an important tool within the multidisciplinary management of refractory post-bariatric leaks, offering improved outcomes for this patient group. Overall, it provides a safe and efficacious rescue option for persistent leaks following bariatric procedures that are resistant to standard endoscopic interventions, with notable success rates and durable benefits. Although limited by sample size, these findings contribute meaningful data to the growing literature supporting OTSC in complex post-bariatric leak scenarios. Further large-scale, prospective studies are needed to validate these results, refine patient selection, determine optimal timing, and assess long-term effectiveness.
